# Selective percutaneous myofasciotomy in persistent toe-walking: Anatomical rationale and surgical technique

**DOI:** 10.1016/j.sopen.2026.08.005

**Published:** 2026-09-12

**Authors:** David Pomarino, Alexander Yakovlevich Nazarkin, Amel Sidi Athmane, Anna Ryzhanok, Bastian Fregien, Kevin M. Rostásy

**Affiliations:** a Pomarino Praxis für Ganganomalien, Hamburg, Germany; b Fakultät für Gesundheit (Department für Humanmedizin), Universität Witten/Herdecke, Witten/Herdecke, Germany

**Keywords:** Persistent toe walking, Equinus deformity, Selective percutaneous myofasciotomy, Ankle dorsiflexion, Minimally invasive surgery

## Abstract

Persistent toe-walking is a gait abnormality characterized by forefoot initial contact and reduced or absent heel strike beyond early childhood. Although often regarded as a transient developmental variant, persistence of this pattern may lead to progressive limitation of ankle dorsiflexion, fixed equinus contracture, and long-term functional impairment. Toe-walking may occur as an isolated idiopathic condition or in association with neurological, neuromuscular, or genetic disorders. Increasing evidence suggests that a subset of patients previously classified as idiopathic harbor underlying hereditary or neuromuscular abnormalities. When left untreated, persistent toe-walking may result in inefficient gait mechanics, increased energy expenditure, compensatory proximal joint adaptations, musculoskeletal pain, and limitations in physical activity.

Conservative management including pyramidal insoles, physiotherapy, stretching, orthoses, serial casting, and botulinum toxin injections may be effective in early or functional stages; however, these approaches frequently fail once fixed myofascial contractures develop. Traditional surgical lengthening procedures can restore ankle dorsiflexion but are associated with risks such as over-lengthening, postoperative weakness, recurrence, and soft-tissue morbidity. Selective percutaneous myofasciotomy represents a minimally invasive alternative based on targeted subcutaneous release of fibrotic myofascial structures while preserving surrounding healthy tissue and normal neuromuscular anatomy.

This article describes the anatomical rationale, indications, and surgical technique of selective percutaneous myofasciotomy for the treatment of ankle equinus in patients with persistent toe-walking. By addressing localized myofascial restrictions with minimal surgical trauma, this technique aims to restore ankle mobility, facilitate gait normalization, and enhance postoperative rehabilitation, while minimizing recurrence and preserving muscle function and improving long-term outcomes in affected children and adolescents.

**Level of evidence:**

Level V.

## Introduction

Toe-walking is a gait pattern characterized by forefoot initial contact during ambulation with absent or reduced heel strike [1]. While commonly observed during early gait development [2], persistence beyond early childhood may be associated with progressive limitation of ankle dorsiflexion, fixed equinus contracture, gait dysfunction, and musculoskeletal symptoms [3], [4], [5]. Persistent toe walking may occur as an isolated condition or in association with neurological, neuromuscular, or developmental disorders.

Although independent ambulation is usually not delayed, the gait pattern is often inefficient and mechanically disadvantageous. Reduced ankle dorsiflexion impairs normal tibial progression during stance, resulting in diminished push-off, increased energy expenditure, and compensatory movements at the knee, hip, and trunk. Over time, these adaptations may contribute to muscle fatigue, pain in the lower extremities or back, frequent stumbling, and limitations in physical activity [6].

Management of persistent toe walking is guided by patient age, ankle range of motion, and the presence and severity of gastrocnemius–soleus contracture. Initial treatment generally consists of conservative measures, including pyramidal insoles, physiotherapy, stretching, orthoses, and serial casting, whereas surgical intervention is reserved for selected patients with more severe deformity or contracture [7].

Surgical treatment of fixed equinus associated with persistent toe walking has traditionally relied on procedures targeting the gastrocnemius–soleus complex and Achilles tendon. Depending on the anatomical level of contracture, the underlying pathology, and the results of the Silfverskiöld test, commonly performed procedures include isolated gastrocnemius recession (e.g., Strayer and Baumann procedures), gastrocnemius–soleus recession (e.g., Vulpius procedure), and Achilles tendon lengthening. These procedures have been shown to improve ankle dorsiflexion and gait in appropriately selected patients; however, each has distinct indications, technical considerations, and potential complications [8].

Gastrocnemius recession is generally reserved for isolated gastrocnemius contracture, whereas Achilles tendon lengthening is typically indicated when both the gastrocnemius and soleus contribute to fixed equinus. Potential complications include over-lengthening, postoperative calf weakness, altered push-off mechanics, recurrence, scar formation, and the need for prolonged postoperative immobilization or rehabilitation. Although minimally invasive modifications have been introduced, most techniques remain based on tendon or aponeurotic lengthening [9], [10].

Selective percutaneous myofasciotomy differs conceptually from these procedures because it aims to release localized fibrotic myofascial restrictions through subcutaneous puncture incisions while preserving the overall continuity of the musculotendinous unit. Rather than replacing established tendon-lengthening procedures, the technique is intended as a minimally invasive alternative for carefully selected patients with fixed myofascial contractures. The purpose of this Technical Note is not to establish superiority over existing procedures but to describe the surgical rationale, operative technique, and practical considerations associated with this approach.

## Pathogenesis

Toe-walking may occur as a manifestation of a variety of underlying neurological and neuromuscular disorders, or present as a persistent gait pattern in the absence of identifiable pathology. The underlying mechanisms differ between these groups but commonly involve altered neuromuscular control and muscle imbalance affecting ankle dorsiflexion. As well as genetic underlying causes are involved [11], [12], [13], [14], [15].

### Genetics

For many years, persistent and idiopathic toe-walking were regarded as conditions without a clearly identifiable cause. Increasing evidence, however, suggests that genetic factors may contribute to the development of toe-walking in a subset of patients. This hypothesis is supported by the frequent observation of toe-walking in multiple members of the same family in about 90% and by the association of the gait pattern with additional phenotypic features suggestive of inherited disorders [16].

Several studies have reported a positive family history in children with persistent or idiopathic toe-walking, indicating a possible hereditary component. In addition to the gait anomaly itself, affected patients may present with other clinical features such as pes cavus, clinodactyly, brachydactyly, pectus deformities, or speech development disorders, further supporting the concept of a systemic rather than purely local musculoskeletal condition [17].

Recent studies using targeted gene panels have demonstrated that a considerable proportion of patients diagnosed with idiopathic toe-walking carry genetic variants classified as pathogenic, likely pathogenic, or of uncertain significance in genes associated with neuromuscular or metabolic disorders. In a retrospective cohort analysis, pathogenic or likely pathogenic variants in genes related to neuropathies or myopathies were identified in a subset of patients previously classified as idiopathic, suggesting that toe-walking may represent an early or isolated manifestation of underlying hereditary disease in some cases [18].

Variants of uncertain significance are frequently detected in this population, and their clinical relevance remains unclear. Nevertheless, the high prevalence of such variants, combined with associated clinical features and family history, indicates that genetic factors may play a contributory role in the pathogenesis of persistent toe-walking. Importantly, the identification of genetic abnormalities may have implications for clinical management, as the response to conservative or surgical treatment may differ depending on the underlying neuromuscular pathology [19].

Genetic testing therefore represents a valuable adjunct in the evaluation of children with persistent toe-walking, particularly in cases with atypical features, progressive deformity, family history, or associated neurological or developmental findings. Recognition of an underlying hereditary condition may allow more accurate diagnosis, individualized treatment planning, and improved counseling of patients and families regarding prognosis and long-term outcomes.

### Neurological conditions

Toe-walking is frequently observed in children with disorders of the central nervous system. It is a common feature in spastic forms of cerebral palsy, where increased muscle tone and impaired selective motor control lead to persistent plantarflexor overactivity. Toe-walking has also been described in children with periventricular leukomalacia, spinal cord abnormalities, and other conditions affecting descending motor pathways. In these disorders, impaired central motor regulation results in abnormal gait patterns that often progress to fixed equinus deformity with growth [20], [21], [22], [23], [24].

### Neuromuscular and muscular disorders

Neuromuscular diseases may also present with toe-walking, either due to muscle weakness, imbalance, or altered muscle activation patterns. Conditions such as Duchenne muscular dystrophy, Charcot–Marie–Tooth disease, and other peripheral neuropathies have been associated with forefoot gait [25], [26].

## Pathological anatomical aspects

The primary pathological changes involve the soft tissues of the posterior compartment of the lower leg and the plantar structures of the foot. Contracture and shortening of the gastrocnemius and soleus muscles are commonly present, often accompanied by tightening of the Achilles tendon. These changes result in persistent plantarflexion of the ankle and restriction of dorsiflexion. This plantarflexed posture corresponds to an equinus foot deformity, characterized by limited heel contact and impaired ankle dorsiflexion.

The gastrocnemius muscle, particularly its medial head, is frequently involved and may demonstrate increased stiffness and reduced extensibility. The soleus muscle is often affected concurrently, contributing to limitation of ankle dorsiflexion both with the knee extended and flexed. In addition to muscular contracture, surrounding connective tissues may undergo adaptive changes in response to altered mechanical loading. Experimental studies have demonstrated that deep fascia is a biologically active tissue capable of extracellular matrix remodeling, with alterations in collagen composition and fascial organization occurring under sustained mechanical tension. These findings suggest that connective tissue adaptation may contribute to the mechanical behavior of the musculotendinous unit, although its precise role in persistent toe walking remains incompletely understood [27].

Persistent toe walking is associated with neuromuscular abnormalities in some patients, while limitation of ankle dorsiflexion is accompanied by secondary biomechanical and soft-tissue adaptations. Biomechanical studies have shown that limitation of ankle dorsiflexion is associated with altered muscle activation patterns and compensatory gait strategies affecting the ankle, knee, and hip, emphasizing that both neural control and mechanical factors contribute to the clinical presentation [28], [29].

Although selective percutaneous myofasciotomy is intended to reduce pathological soft-tissue tension through targeted release of myofascial structures, the relative contributions of muscle, fascia, tendon, and neural mechanisms to its clinical effect remain uncertain. Further studies are required to clarify the biological mechanisms underlying this approach and to determine which tissue components contribute most to correction of equinus deformity.

The severity of these anatomical changes correlates with the duration of toe-walking and the degree of limitation in ankle dorsiflexion (Fig. 1).

**Fig. 1:**
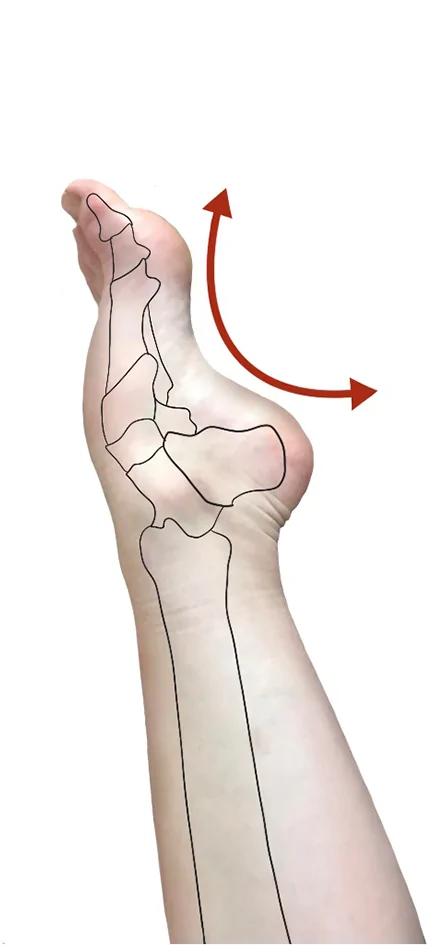
Equinus foot posture demonstrating persistent plantarflexion and restricted ankle dorsiflexion. (Illustration created by Antonia Graaf).

## Clinical features

Persistent toe walking is characterized by a gait pattern in which initial foot contact occurs at the forefoot rather than with a physiological heel strike and roll-over. Clinically, toe walking is typically considered relevant when it is present for a substantial proportion of daily ambulation and does not resolve spontaneously with growth. While many children exhibit transient toe walking during early gait development, persistence beyond early childhood is associated with functional limitations and may warrant further evaluation.

A central clinical feature in patients presenting for treatment is restricted mobility of the upper ankle joint, most commonly manifesting as limited ankle dorsiflexion. This limitation is frequently associated with an equinus position of the foot during stance and gait. Using the neutral zero method, normal upper ankle joint mobility is commonly referenced as 20–0–50° [30], and patients indicated for intervention typically demonstrate a clinically relevant reduction in dorsiflexion compared with this reference range. Reduced ankle mobility contributes to an abnormal gait pattern, impaired push-off mechanics, and compensatory strategies during walking.

In addition to gait abnormalities, patients may present with functional complaints such as frequent tripping or stumbling, reduced endurance, and difficulties with balance. In older children, adolescents, and adults [31], persistent toe walking may be associated with musculoskeletal discomfort, including pain in the feet, calves, knees, or lower back. Social and psychological factors, such as self-consciousness or teasing due to the conspicuous gait pattern, have also been reported as contributing reasons for seeking treatment.

“Clinical examination typically demonstrates limited ankle dorsiflexion associated with organic contracture of the gastrocnemius and/or soleus muscles. In some patients, stiffness of the plantar structures of the foot may contribute to restricted forefoot mobility. The severity of clinical findings varies considerably between individuals.

### Illustrative clinical cases

To illustrate the clinical application of selective percutaneous myofasciotomy, three representative patients treated at our institution are presented.

#### Case 1

A patient presented with persistent toe walking since early childhood associated with progressive worsening of gait, pain during walking, and lumbar hyperlordosis. Preoperative examination demonstrated marked restriction of ankle dorsiflexion (left: 0–10–50°, right: 0–20–50°). Selective percutaneous myofasciotomy was performed because of the severity of the deformity. At follow-up, the patient no longer demonstrated toe walking during provocation testing. Lumbar hyperlordosis had resolved, ankle dorsiflexion improved bilaterally to 15–0–50°, and walking-related pain was reduced.

#### Case 2

A patient with persistent toe walking since the onset of independent walking presented with progressive gait impairment and ankle pain. Preoperative ankle dorsiflexion measured 0–10–50° bilaterally. Following selective percutaneous myofasciotomy and postoperative treatment with orthoses and night splints, the patient achieved spontaneous plantigrade gait. Ankle dorsiflexion improved bilaterally to 25–0–50°, and the overall gait pattern was markedly improved.

#### Case 3

A 15-year-old patient with persistent toe walking since the onset of walking presented with severe forefoot gait and difficulty walking on the heels. Preoperative ankle dorsiflexion measured 5–0–50° on the left and 0–0–50° on the right. After selective percutaneous myofasciotomy, the patient no longer demonstrated toe walking during provocation testing. Bilateral ankle dorsiflexion improved to 15–0–50°, accompanied by reduced walking-related complaints.

Representative intraoperative photographs illustrating the key surgical steps of selective percutaneous myofasciotomy are shown in Fig. 2, Fig. 3. These images are provided to demonstrate the surgical technique and are not intended to correspond to the illustrative cases described above. Representative preoperative and postoperative clinical photographs demonstrating correction of equinus deformity are presented in Fig. 4, Fig. 5 and are likewise included for illustrative purposes.

**Fig. 2:**
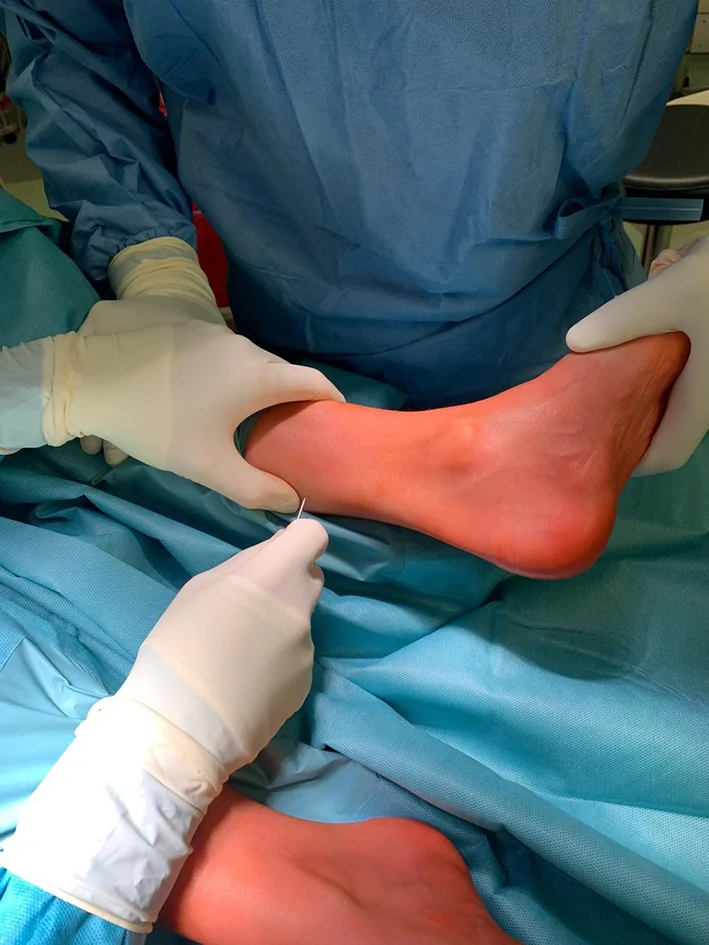
Representative intraoperative identification of the targeted myofascial structures before selective percutaneous myofasciotomy.

**Fig. 3:**
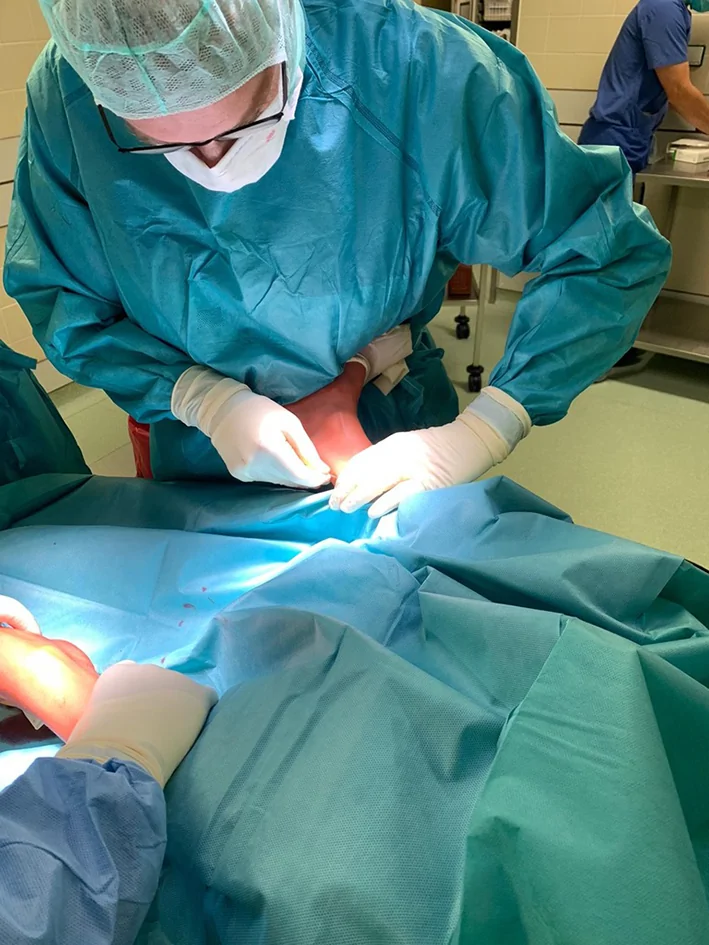
Representative intraoperative performance of selective percutaneous myofasciotomy through a minimal skin incision.

**Fig. 4:**
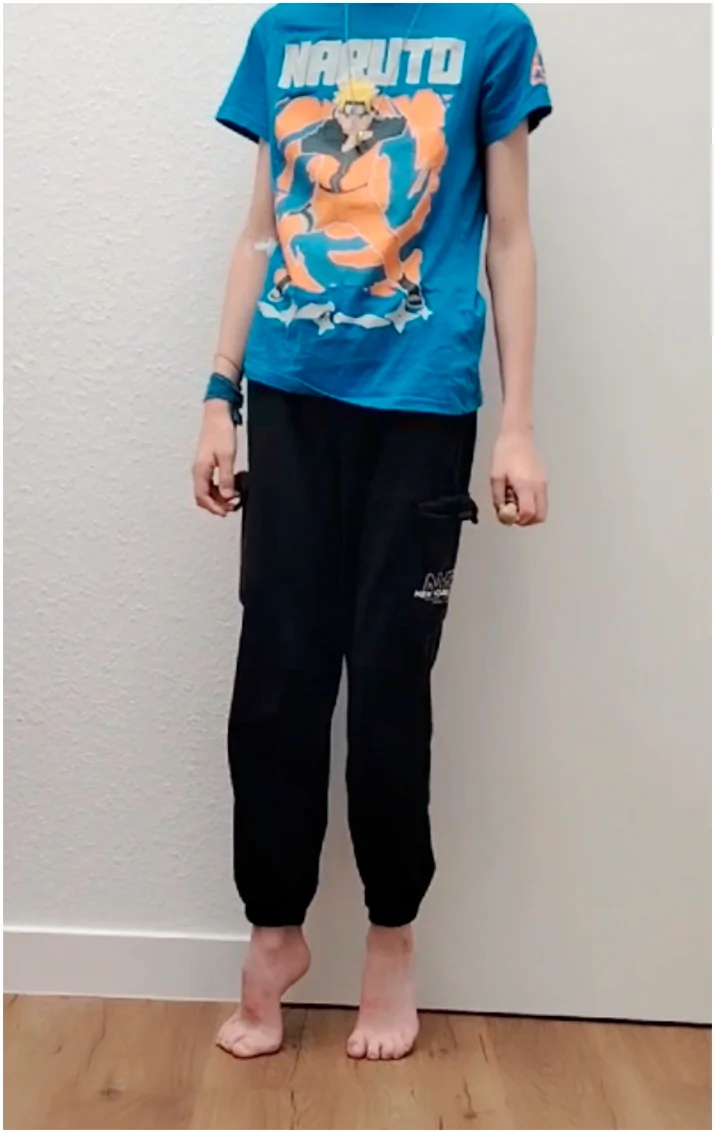
Representative clinical appearance before selective percutaneous myofasciotomy. (Preoperative bilateral toe walking).

**Fig. 5:**
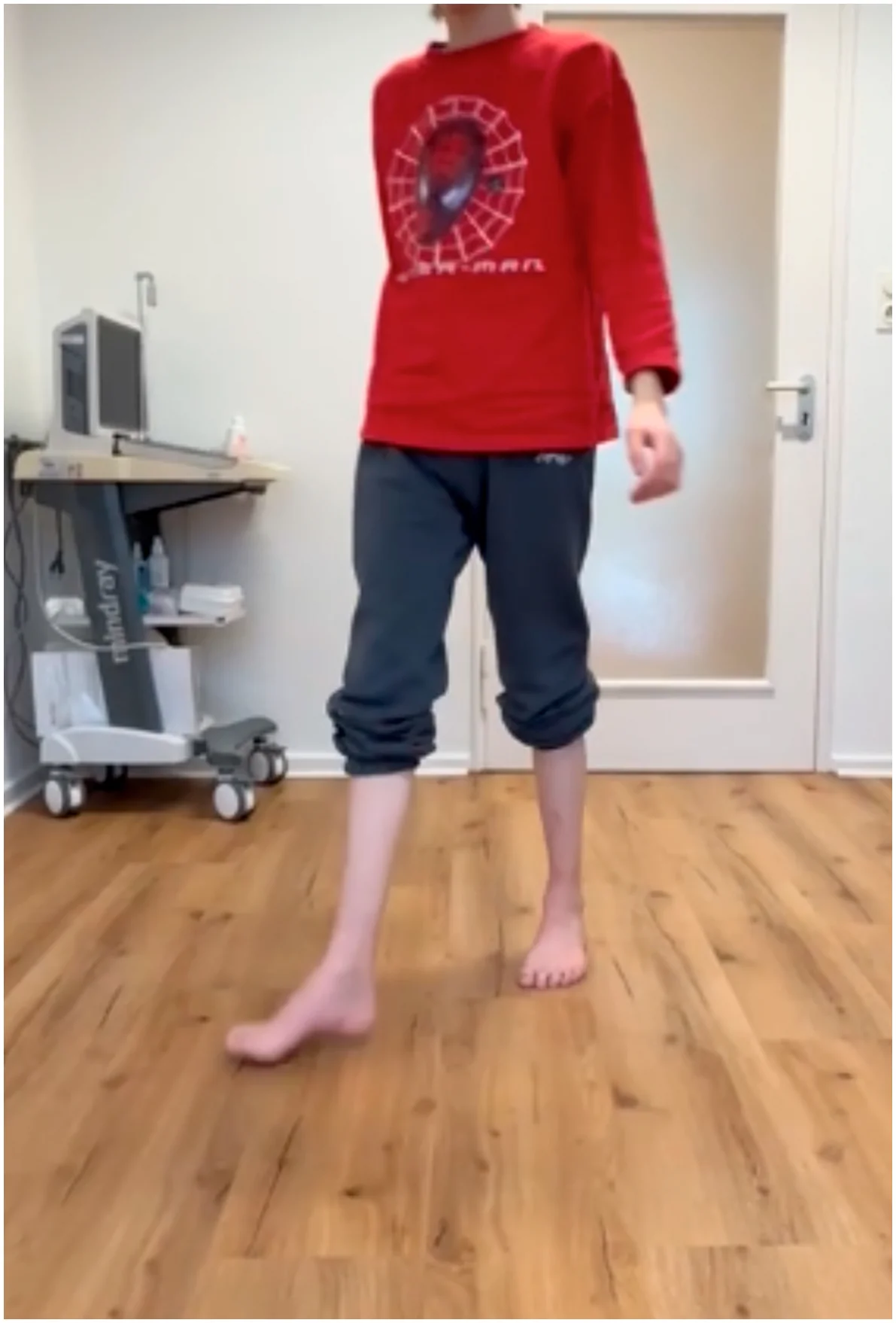
Representative clinical appearance after selective percutaneous myofasciotomy. (Postoperative restoration of plantigrade stance).

## Treatment

The primary therapeutic goal in the treatment of toe-walking with equinus deformity is the restoration of sufficient ankle dorsiflexion to allow physiological heel contact during stance and a more normalized gait pattern. Persistent limitation of dorsiflexion is most commonly caused by fixed soft-tissue contractures of the posterior lower leg and plantar structures, which cannot be adequately addressed once they have progressed beyond a functional stage.

Selective percutaneous myofasciotomy is based on the concept that equinus in toe-walking is predominantly a soft-tissue–driven deformity resulting from localized fibrotic and myofascial shortening rather than primary osseous malalignment. The rationale of this approach is to target these pathological soft-tissue constraints in a minimally invasive manner, with the aim of improving muscle elasticity and increasing the functional range of motion of the ankle joint.

By addressing the anatomical substrate responsible for fixed equinus, selective percutaneous myofasciotomy seeks to improve upper ankle joint mobility while preserving overall muscle function and minimizing surgical trauma. This approach is intended to facilitate subsequent gait normalization and to enhance the effectiveness of postoperative rehabilitation.

## Conventional management strategies for persistent toe walking

Current treatment strategies for persistent toe walking are primarily guided by patient age, ankle range of motion, presence of fixed contracture, and foot morphology. In children without fixed contracture, nonsurgical management such as physiotherapy, stretching, and bracing is commonly recommended [32]. However, available evidence suggests that these interventions result in only minimal improvements in ankle dorsiflexion, typically in the range of 1–2°, and rarely lead to sustained normalization of gait [33].

A retrospective case series of 193 children reported that treatment with pyramidal insoles was successful in approximately 90% of cases, with significant improvement in ankle dorsiflexion. Better outcomes were observed when treatment was initiated at a younger age, whereas older age at presentation and declining ankle range of motion during therapy were associated with treatment failure [34].

Serial casting has been shown to improve ankle dorsiflexion and gait parameters in children with toe walking, with normalization of several functional measures reported at mid-term follow-up. However, outcomes vary across studies, and long-term durability remains inconsistent, particularly in patients with established contractures [22], [35], [36].

Botulinum toxin A has been investigated as a treatment option for idiopathic toe walking; however, high-level evidence does not demonstrate a clinically meaningful benefit. Its use has not been shown to result in improvements in heel-strike gait, or overall toe-walking severity compared with established conservative measures, while being associated with a notable rate of adverse events. Consequently, current evidence does not support the routine use of botulinum toxin A in the management of idiopathic toe walking [37].

In cases of fixed equinus deformity, surgical lengthening of the gastrocnemius–soleus complex and, when indicated, plantar fascia release are widely accepted treatment options. These procedures can effectively improve ankle dorsiflexion [38]; however, they are associated with inherent risks, including over-lengthening, postoperative weakness, recurrence, and incomplete normalization of gait mechanics. Furthermore, these approaches typically involve open or mini-open techniques with greater soft-tissue disruption [39], [40].

Taken together, these limitations highlight the need for alternative treatment strategies that address fixed soft-tissue contractures while minimizing surgical trauma and preserving muscle function.

### Surgical technique: selective percutaneous myofasciotomy

Selective percutaneous myofasciotomy (SPM), also referred to as selective myofibrofasciotomy (SMFT) or the Ulzibat method, is a minimally invasive soft-tissue procedure designed to address fixed myofascial contractures contributing to equinus deformity in toe-walking patients. The technique is based on the selective subcutaneous release of fibrotic muscle fibers and fascia while preserving surrounding healthy tissue and normal neuromuscular structures. By targeting pathological myofascial restrictions rather than performing extensive tendon lengthening, SPM aims to restore joint range of motion with minimal surgical trauma and a short recovery period. The following section provides a detailed description of the indications, operative setup, surgical steps, and postoperative management of this procedure, with particular emphasis on its application in the treatment of ankle equinus associated with persistent toe walking.

1. Indications and contraindications

Indications

• Presence of persistent toe walking
• limited mobility of the upper ankle joint
• Presence of fixed (organic) muscle contracture affecting the gastrocnemius–soleus complex and/or plantar fascia
• Insufficient effect of conservative therapy and rehabilitation, or improvement that remains incomplete due to organic contracture

Contraindications

• Absence of fixed (organic) muscle contracture (i.e., purely functional limitation)
• Preserved ankle dorsiflexion without structural restriction
• Inability to safely perform the procedure under general anesthesia on an outpatient basis
• Severe general condition precluding outpatient surgical management
• Presence of a coagulopathy

2. Anesthesia and patient positioning

Anesthesia

• Selective percutaneous myofasciotomy is performed under general anesthesia.
• The procedure is designed to be carried out in outpatient settings, provided general anesthesia can be safely administered.

Patient positioning

• For treatment of equinus deformity at the ankle, the patient is positioned supine.
• During the procedure, an assistant performs controlled dorsiflexion of the foot to create tension in the posterior compartment muscles of the lower leg.
• Correct positioning of the foot relative to the lower leg is emphasized, avoiding valgus or varus alignment during manipulation.
• Muscle tension created by positioning and passive movement is used to facilitate selective subcutaneous dissection of fibrotic muscle fibers and fascia.

3. Instruments

Selective percutaneous myofasciotomy is performed using a specialized scalpel specifically designed for subcutaneous, selective dissection of fibrotic muscle fibers and fascia (Fig. 6). The instrument allows the procedure to be carried out through a skin puncture of less than 1 mm, eliminating the need for open incisions. The shape and cutting edge of the blade are engineered to enable precise, controlled dissection of fibrotic tissue while minimizing trauma to surrounding healthy muscle fibers and adjacent structures.

**Fig. 6:**
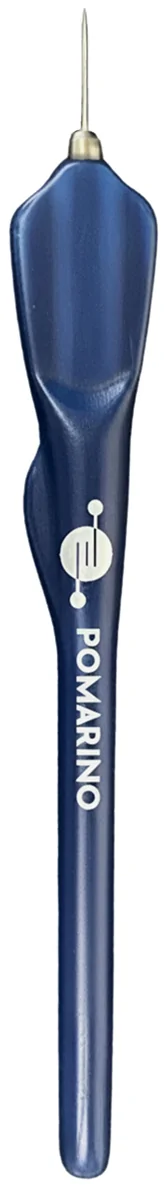
Specialized scalpel used for selective percutaneous myofasciotomy (Illustration created by Antonia Graaf).

The technique does not require extensive surgical instrumentation. Standard surgical equipment for skin preparation, antiseptic wound care, and basic hemostasis is sufficient. No retractors or visualization instruments are necessary, as the procedure is performed in a closed, subcutaneous manner. The limited instrumentation contributes to the short operative time and low soft-tissue trauma associated with the method.

Because the procedure is performed without direct visual control of the blade within the tissues, surgeon experience and familiarity with the instrument are essential to ensure selective release of fibrotic structures and to minimize the risk of iatrogenic injury to neurovascular elements.

4. Step-by-step technique

In the ankle joint, pathological restriction most commonly affects ankle dorsiflexion, resulting in an equinus deformity. This limitation is primarily caused by fixed (organic) contractures of the posterior compartment muscles of the lower leg. Selective percutaneous myofasciotomy is performed in a stepwise manner, targeting only the muscles and fascia contributing to the restriction.

### Gastrocnemius muscle SMFT

Indication

• Limitation of ankle dorsiflexion (< 5°) due to contracture of the gastrocnemius muscle.

Patient position and tensioning

• The patient is positioned supine.
• An assistant performs controlled dorsiflexion of the foot to create tension in the posterior muscle group of the lower leg.
• Correct foot positioning is essential, avoiding valgus or varus alignment relative to the lower leg.

Technique

• A skin puncture is made at the border between the middle and lower thirds of the lower leg on the medial side (Fig. 7).

**Fig. 7:**
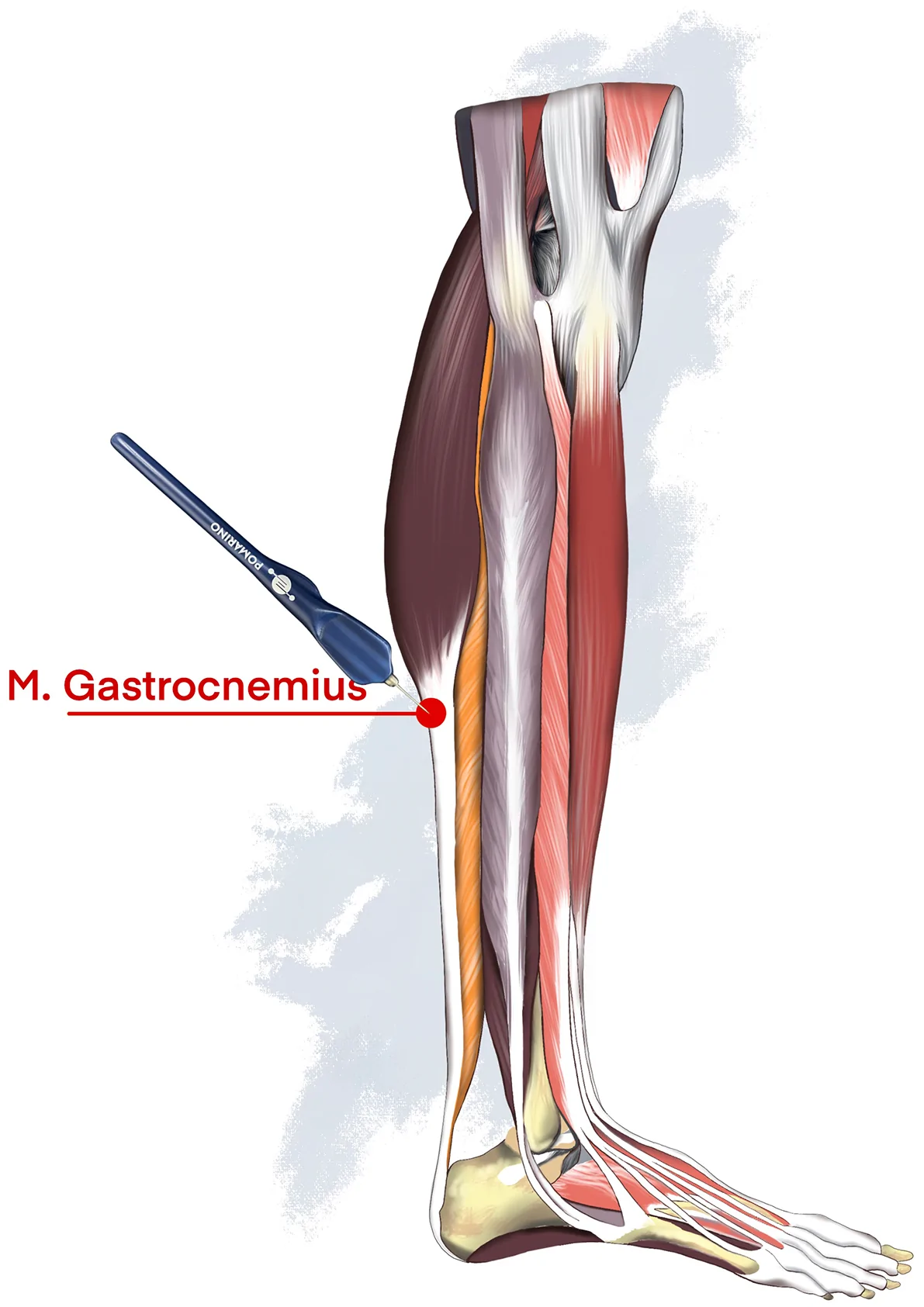
Medial approach and subcutaneous release of the gastrocnemius muscle (Illustration created by Antonia Graaf).
• The scalpel is introduced subcutaneously to the level of the fibrosed fascia of the gastrocnemius muscle.
• Selective superficial dissection is performed across the width of the fibrotic fascia, without extending into healthy muscle tissue.

Intraoperative control and endpoint

• The surgeon and assistant continuously monitor ankle range of motion.
• If dorsiflexion exceeds 5°, the procedure is stopped.
• Criterion for success: elimination of flexion contracture of the ankle joint.

### Soleus muscle SMFT

Indication

• Persistent limitation of ankle dorsiflexion involving the soleus muscle.

Patient position and tensioning

• The patient remains in the supine position.
• The assistant dorsiflexes the foot, creating tension in the posterior calf muscles.
• Neutral alignment of the foot is maintained throughout the procedure.

Technique

• A skin puncture is made at the border between the middle and lower thirds of the lower leg on the medial side, similar to the gastrocnemius approach (Fig. 8).

**Fig. 8:**
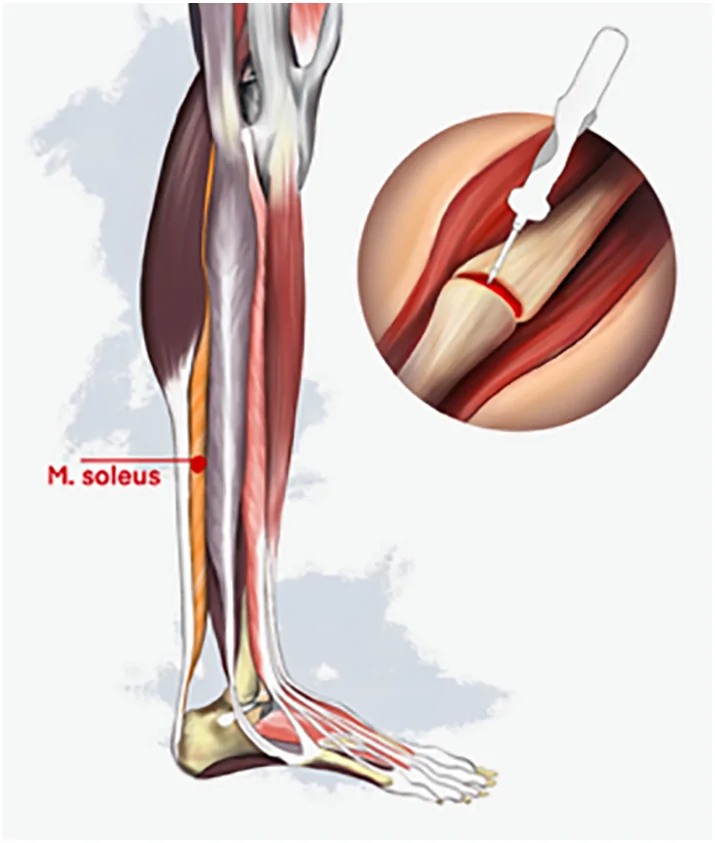
Subcutaneous selective release of the soleus muscle (Illustration created by Antonia Graaf).
• The scalpel is advanced subcutaneously to the level of the fibrosed medial edge of the soleus muscle.
• Selective incision of fibrotic tissue is performed, without penetrating beyond the limits of the muscle.

Endpoint

• Criterion for success: elimination of ankle flexion contracture.

### Plantar Fascia SMFT

Indication

• Clinically identified restriction of forefoot mobility for which release of the plantar fascia is considered appropriate.

Patient position and tensioning

• The patient is positioned supine.
• The assistant dorsiflexes the foot while simultaneously extending the toes to tension the plantar aponeurosis.

Technique

• A skin puncture is made at the attachment of the plantar aponeurosis to the calcaneal tuberosity (Fig. 9).

**Fig. 9:**
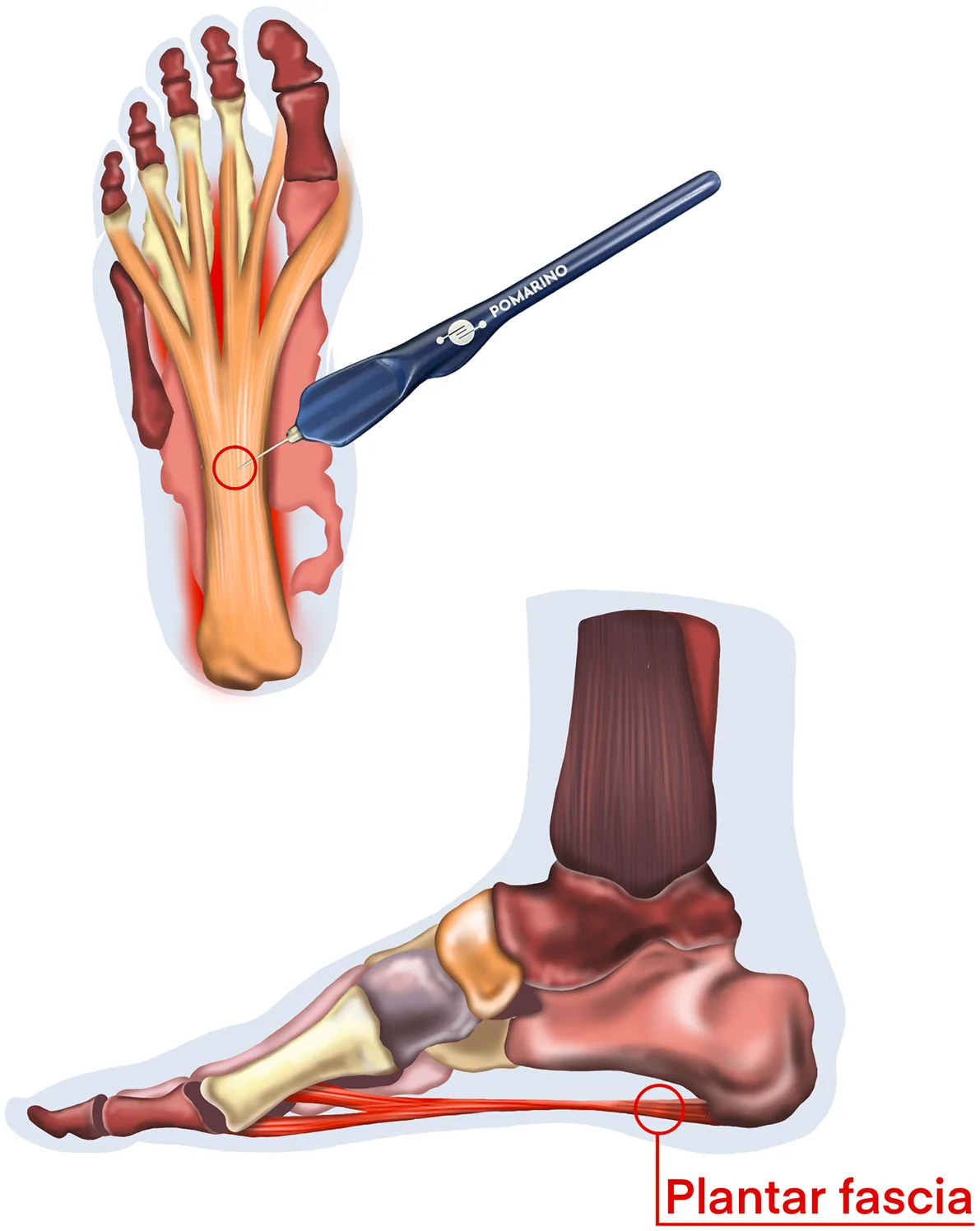
Selective percutaneous release of the plantar fascia (Illustration created by Antonia Graaf).
• The scalpel is placed on the fibrotic plantar fascia and selectively dissected medially away from the body.

Endpoint

• Criterion for success: mobilization of the anterior part of the foot.

5. Postoperative management

Postoperative management following selective percutaneous myofasciotomy is generally straightforward because of the minimally invasive nature of the procedure. Patients are typically treated on an outpatient basis and discharged on the day of surgery or after a short observation period.

Immediate postoperative care

• Local antiseptic care of the puncture sites is performed for 2–3 days.
• Patients may begin full weight-bearing with orthoses on the first postoperative day, provided there are no contraindications.
• Small postoperative hematomas or swelling are managed conservatively with local measures and, when indicated, physiotherapy.

Follow-up and rehabilitation

• The first postoperative follow-up is performed approximately 7 days after surgery, during which instructions for using aids are reinforced.
• During the first 2 weeks, patients are monitored for postoperative swelling or hematoma formation.
• Prescribed aids are continued according to the individual clinical situation. Around 4 weeks after surgery, these are gradually discontinued if adequate clinical progress has been achieved.
• Progressive rehabilitation is encouraged throughout recovery to improve ankle mobility, gait pattern, and functional performance.

Return to activity

• Participation in sports is generally restricted during the early postoperative period and may be resumed approximately 6 weeks after surgery, depending on clinical recovery and functional assessment.
• A further follow-up is performed at approximately 10–12 weeks to evaluate overall functional outcome.

Postoperative management should always be individualized according to patient age, the severity of the deformity, associated conditions, and clinical progress.

6. Complications / limitations / pearls & pitfalls

Complications

Although selective percutaneous myofasciotomy is designed to minimize soft-tissue disruption through a minimally invasive approach, it remains a surgical procedure with inherent risks. Potential complications include:

• Incomplete release of the pathological myofascial structures, resulting in persistent limitation of ankle dorsiflexion or residual equinus deformity.
• Overcorrection or excessive release, which may lead to muscle weakness, altered gait mechanics, or reduced plantarflexion strength.
• Iatrogenic injury to adjacent neurovascular structures, as the procedure is performed through small skin punctures without direct visualization. Careful knowledge of regional anatomy and meticulous surgical technique are essential to minimize this risk.
• Postoperative hematoma or localized bleeding may occur, although these are typically managed conservatively.
• Superficial wound complications, including delayed healing or infection of the puncture sites, are possible despite the minimal skin incision.
• Recurrence of equinus deformity, particularly in growing children or in patients with progressive underlying neuromuscular disorders.
• Persistent gait abnormalities or incomplete functional improvement, despite improved ankle dorsiflexion, particularly when long-standing compensatory gait patterns or neurological disorders are present.

Limitations

• The technique is ineffective in the absence of fixed (organic) muscle contracture and should not be used in cases of purely functional or neurogenic contracture.
• Accurate differentiation between functional and fixed contracture is essential; failure to do so may lead to insufficient clinical improvement.
• Recurrence of contracture is possible, particularly in pediatric patients, as continued skeletal growth may outpace soft-tissue adaptation.
• The procedure relies heavily on surgeon skill and anatomical knowledge, as tactile feedback and palpation guide the selective dissection.
• Although the preliminary clinical experience with selective percutaneous myofasciotomy is encouraging, the current evidence remains limited. Large prospective comparative studies with standardized outcome measures and long-term follow-up are required to further evaluate the safety, efficacy, and appropriate indications for this technique before widespread adoption can be recommended.

Pearls

• Careful patient selection based on the presence of organic contracture and limited joint range of motion is critical for successful outcomes.
• Treating the lower limb as an integrated biomechanical system improves outcomes, particularly when multiple joint contractures coexist.
• Starting with the most pronounced contracture helps restore biomechanical balance and facilitates subsequent correction of less severe deformities.
• Continuous intraoperative monitoring of joint range of motion allows the surgeon to avoid overcorrection and unnecessary tissue disruption.
• Careful identification of anatomical landmarks, controlled positioning of the foot and ankle, and selective release of only the pathological myofascial structures help maintain intraoperative safety and reduce the risk of neurovascular injury or excessive tissue release.
• The procedure should be performed only by surgeons familiar with the regional anatomy and the specialized instrumentation, as the absence of direct visualization requires reliance on anatomical knowledge, tactile feedback, and continuous assessment of ankle dorsiflexion throughout the procedure.
• The minimally invasive nature of SMFT allows for repeat procedures, if required, without significant additional morbidity.

Pitfalls

• Performing SMFT in patients with purely functional contracture or spasticity without fibrosis may result in no meaningful improvement.
• Over-dissection or imprecise scalpel movement may lead to muscle weakness or biomechanical imbalance, particularly in muscles with critical functional roles (e.g., posterior thigh muscles).
• Failure to use postoperative orthotic support or activity modification, when indicated, may increase the risk of recurrence.
• Ignoring synergistic and antagonistic muscle relationships during surgical planning may compromise functional outcomes.

## CRediT authorship contribution statement

**David Pomarino:** Writing – original draft, Project administration, Investigation, Data curation, Conceptualization. **Alexander Yakovlevich Nazarkin:** Writing – original draft, Validation, Methodology, Formal analysis, Data curation, Conceptualization. **Amel Sidi Athmane:** Writing – original draft, Validation, Project administration, Formal analysis, Data curation. **Anna Ryzhanok:** Writing – original draft, Validation, Investigation, Data curation. **Bastian Fregien:** Writing – review & editing, Visualization, Validation, Supervision, Methodology, Formal analysis. **Kevin M. Rostásy:** Writing – review & editing, Validation, Supervision, Formal analysis, Data curation.

## Ethical statement

This manuscript describes a surgical technique and does not involve the collection, analysis, or reporting of individual patient data. Therefore, formal ethical approval and informed consent were not required.

## Declaration of Generative AI and AI-assisted technologies in the writing process

The authors declare the use of the generative artificial intelligence tool ChatGPT (OpenAI) during the preparation of this manuscript for assistance with language editing and drafting of selected text sections. The authors verified the accuracy, validity, and originality of all content and ensured that all interpretations, references, and conclusions are their own. No generative AI tool was involved in data analysis, clinical decision-making, or authorship of this work.

## Funding

This research received no specific grant from any funding agency in the public, commercial, or not-for-profit sectors.

## Declaration of competing interest

The Author(s) declare(s) that there is no conflict of interest.

## Glossary

Persistent toe walking (PTW): Toe-walking that continues beyond early childhood, typically after 3 years of age.
Equinus deformity: Fixed plantarflexed position of the ankle with limited or absent dorsiflexion.
Dorsiflexion: Upward movement of the foot at the ankle joint toward the tibia.
Plantarflexion: Downward movement of the foot away from the tibia.
Gastrocnemius–soleus complex (triceps surae): The primary calf muscle group responsible for ankle plantarflexion.
Selective percutaneous myofasciotomy (SPM): Minimally invasive surgical technique involving targeted subcutaneous release of fibrotic myofascial structures to improve joint mobility; also referred to as the Ulzibat method.
Myofascial contracture: Pathological shortening and fibrosis of muscle and surrounding fascia leading to restricted joint motion.
Serial castin: Progressive immobilization using sequential casts to gradually increase joint range of motion.
Orthoses: External supportive devices (e.g., braces or splints) used to maintain alignment and improve function.
Neutral zero method: Standardized system for measuring joint range of motion using 0° as the anatomical neutral position.
